# Holographic Recording Performance of Acrylate-Based Photopolymer under Different Preparation Conditions for Waveguide Display

**DOI:** 10.3390/polym13060936

**Published:** 2021-03-18

**Authors:** Zhongwen Shen, Yishi Weng, Yuning Zhang, Chuang Wang, Ao Liu, Xiaohua Li

**Affiliations:** Joint International Research Laboratory of Information Display and Visualization, School of Electronics and Engineering, Southeast University, Nanjing 210018, China; 220151185@seu.edu.cn (Z.S.); wings@seu.edu.cn (Y.W.); 220191379@seu.edu.cn (C.W.); 13813929480@139.com (A.L.); lxh@seu.edu.cn (X.L.)

**Keywords:** photopolymer, gratings, optical waveguides, holography

## Abstract

This work proposes a green light-sensitive acrylate-based photopolymer. The effects of the preparation conditions for the waveguide applied volume holographic gratings (VHGs) were experimentally investigated. The optimum preparation conditions for holographic recording were revealed. After optimization, the peak of VHG diffraction efficiency reached 99%, the diffractive wavelength bandwidth increased from 13 nm to 22 nm, and the corresponding RIM was 0.06. To prove the wide application prospect of the acrylate-based photopolymer in head-mounted augmented reality (AR) displays, green monochromatic volume holographic waveguides were fabricated. The display results showed that the prototype was able to achieve a 28° diagonal FOV and possessed a system luminance of 300 cd/m^2^.

## 1. Introduction

Volume holographic waveguide is considered to be a potential optical solution for augmented reality (AR) glasses because of its high optical efficiency, low cost, small size and light weight [1,2,3,4]. Traditional holographic interferometry is used to fabricate the volume holographic waveguide for head-mounted AR display [2]. According to the vector circle analysis method, volume holographic gratings (VHGs) with different parameters can be prepared by adjusting the angles between the object light beam and the reference light beam [5,6], which requires the dynamic response range and refractive index modulation (RIM) of the holographic recording materials to be large enough [7]. Additionally, the field of view (FOV) and light efficiency of the volume holographic waveguide are also dependent on the RIM of the holographic recording materials [8,9,10].

Currently, there are many kinds of holographic recording materials, including dichromate gelatin, silver salt, photoresist, photopolymer, etc. [11]. Among them, photopolymer is widely used in holographic waveguide displays [12,13,14,15], optical holographic storage [16], holographic anti-counterfeiting [17], optical communication [18], holographic solar concentrators [19], and holographic beam-shaping diffractive diffusers [20] due to its advantages, which include high diffraction efficiency, high sensitivity, low price and easy preparation. To date a variety of photopolymers (PQ/PMMA, PVA/AA, etc.) with excellent properties have been proposed and studied [21,22,23]. The host material of PQ/PMMA photopolymer is methyl methacrylate (MMA), which is not easy to shrink in volume following polymerization, so PQ/PMMA-based photopolymer is suitable for optical holographic storage [24]. Additionally, the host material of PVA/AA-based photopolymer is polyvinyl alcohol, which has the advantages of good film-forming ability, high photosensitivity, and good diffraction efficiency (up to 100%) [21]. However, the material is soluble in deionized water and susceptible to moisture. To maintain excellent holographic performance, it needs to be encapsulated after exposure. The film-forming materials of acrylate-based photopolymers are generally soluble in organic solvents, and are thus not easily affected by damp, giving them the advantage of environmental stability. Therefore, acrylate-based photopolymers are one of the most promising holographic recording materials that are suitable for the waveguide display [25].

To date, the influences of preparation conditions on the holographic properties of acrylate-based photopolymers have not been systematically studied. Therefore, it is urgent to develop an optimized preparation method for fabricating holographic waveguides with acrylate-based photopolymers for waveguide display.

Erythrosin B (EB) is an excellent photosensitizer and is commonly used in photopolymerization initiation systems of photopolymer [26,27]. In this paper, we studied the holographic recording characteristics of green sensitive acrylate photopolymers doped with EB. To optimize the holographic performance of the waveguide applied VHGs, the effects of preparation conditions on the peak diffraction efficiency, diffractive wavelength bandwidth and RIM were studied in this paper. Monochromatic green holographic waveguide samples were also fabricated by means of laser exposure method, which proves that the acrylate photopolymer has a broad application prospect in the field of holographic waveguide display.

## 2. Experimental Setup

### 2.1. Preparation of the Photopolymer

The acrylate-based photopolymer consisted of four main components, polyvinyl acetate, N-vinyl carbazole (NVC), tetrahydrofurfuryl acrylate, 2-phenoxyethy acrylate purchased from Wraio, Guangzhou, China, which are the key components for polymerization in the photo-initiation process. (2,2′-bis(2-dichlorophenyl)-4,4′,5,5′-tetraphenyl-1,2′-biimidazole (BCIM)) purchased from Tronly, Changzhou, China served as the free radical photo-initiator, which is related to the monomers. The dye EB purchased from Sigma-Aldrich, Burlington, MA, USA, is sensitive to green wavelength bands and has an absorption peak at 533 nm. The concentration ratios of the components are listed in Table 1.

After weighing all the components listed above, they were mixed in a glass beaker using a magnetic stirrer at a rate of 1000 r/min for about 1 h, mixing the components thoroughly to form a uniform solution. Then, the dissolved photopolymer solution was coated onto a 1-mm-thick BK7 transparent glass plate using an electric wet film coater (AB4120 AFA, TQC, The Netherlands). The glass plate coated with the wet photopolymer solution was dried in a dark clean room for several hours to obtain a 12-um-thick photopolymer holographic dry plate. Since all the components were evenly dispersed in the solvent, the photopolymer holographic dry plate samples appeared clear without any impurities under the red safety light.

### 2.2. Holographic Setup for Fabricating the Holographic Waveguide

**Figure 1:**
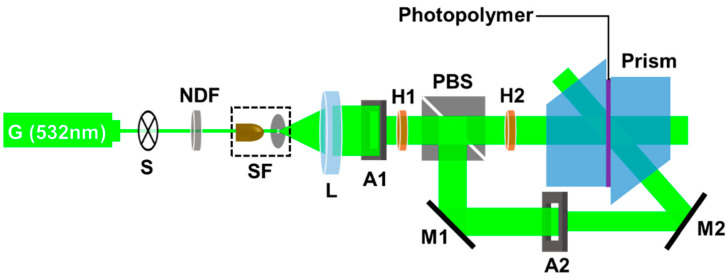
Experimental setup for fabricating the holographic waveguides. S: shutter; NDF: neutral density filter; M: mirror; SF: spatial filter; L: lens, A: aperture; PBS: polarization beam splitter; H: half wave plate.

The holographic setup for the waveguide applied VHGs is shown in Figure 1. The light beams coming from the single longitudinal solid-state lasers (532 nm, Cobolt Corporation, Norway, Sweden) firstly pass through the electric shutter (GCI-7101M) and neutral density filter (GCO-0701M, Daheng Optics, Beijing, China). The shutter is used to control the switching on and off of the optical path. The total light intensity of the reference and object light beams were adjusted by the neutral density filter, and the exposure dosage was adjusted by varying the exposure time. Firstly, the light beams were filtered and expanded using a spatial filter (GCO-0112M, Daheng Optics, Beijing, China) and collimated with the double-glued achromatic lens (GCL-010615, Daheng Optics, Beijing, China). The apertures were used to adjust the spot size and shape of the light beams. To ensure the consistency of the reference light beam and the intensity ratio and polarization state of the object light beam, two half wave plates H1, H2 (GCL-0607, Daheng Optics, Beijing, China) and a polarization beam splitter (GCC-402103, Daheng Optics, Beijing, China) were used. The angle between the reference beam and the object beam was changed using mirrors M1 and M2. Two K9 trapezoid prisms were used to couple the interference of the reference and object light beams and export them for recording by VHG-couplers. Specifically, firstly, the reference light beam was normally incident to the right angle plane of the prism, and the object beam was incident to the prism’s hypotenuse, then both of them were incident to the photopolymer. Considering that the photopolymer has a similar refractive index to the prism, the angle of the reference and object light beams was set as 60°. To avoid the air gap between the prism and photopolymer, refractive index matching oil (*n* = 1.52) was used to fill the gap.

The in-coupling and out-coupling VHGs for the holographic waveguide were mirror symmetrical [4]. When the first grating was done, the second grating was recorded on the other end of the photopolymer sample using a mirror operation. To analyze the influences of exposure parameters on holographic characteristics of the acrylate-based photopolymer, we fabricated waveguide applied VHG samples under different exposure conditions.

### 2.3. Post-Treatment Process for Fabricating Holographic Waveguide

**Figure 2:**
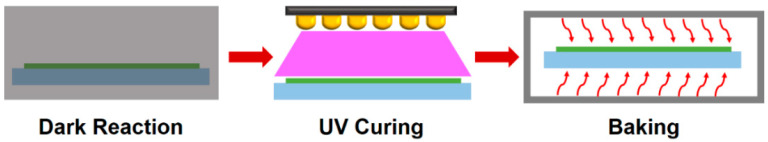
Post-treatment for fabricating holographic waveguides.

As shown in Figure 2, post-treatment must be carried out after holographic recording. To ensure that the monomers reacted completely and polymerized, the samples were placed for a period of 1 to 5 min in a dark room. Then the samples were illuminated under 365 nm ultraviolet (UV) light to fix the internal grating fringes. Finally, in order to achieve higher optical efficiency of waveguide applied VHGs, we placed the samples in a vacuum drying chamber and heated them for a period of time at a certain temperature.

### 2.4. Holographic Characterization Methods of Waveguide Applied VHGs

**Figure 3:**
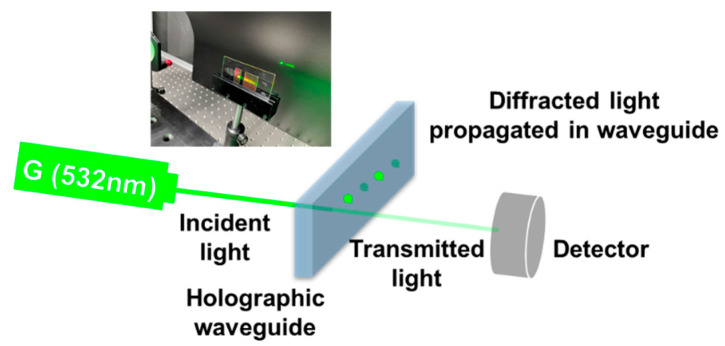
Measurement of the optical path in order to calculate the maximum diffraction efficiency of the waveguide applied VHGs. The light intensity was measured using a laser power meter (OPHIR Vega). The actual diffraction phenomena of the waveguide applied VHG are also shown in the figure.

Both the exposure parameters (exposure dosage, exposure intensity, the intensity contrast of reference and object light beams) and post-treatment parameters (dark reaction time, UV curing time, baking temperature and time) have a significant effect on the performance of holographic optical elements (HOEs) prepared by the photopolymer. Therefore, we analyzed the diffraction efficiency, diffractive wavelength bandwidth, and RIM evolution of waveguide applied VHGs under different combinations of the above-mentioned preparation conditions. The diffraction efficiency plays an important role in describing the holographic performance, which was investigated first. In Figure 3, the incident intensity I_in_ and the transmitted intensity I_o_ of the probe beam were measured using a laser power meter (OPHIR Vega, Jerusalem, Israel). The diffraction efficiency was calculated as

η = (1 − I_o_/I_in_) × 100%. (1)

The diffractive efficiency curve under different wavelengths ranging from 450 nm to 600 nm was obtained using a visible ultraviolet spectrophotometer (TU-1901, Persee Corporation, Beijing, China). On the basis of the designed grating parameters, the measured peak diffraction efficiency and the diffraction efficiency curve, we were able to deduce the RIM of the samples using the rigorous finite element method (FEM) model for the VHGs. The FEM model setup was introduced in our previous work [4]. The refractive index distribution equation of the VHGs was calculated as

(2) $$n=n_{0}+\Delta \mathrm{ncos}\left[\frac{2\pi }{\Lambda }(\mathrm{xsin}\left(\varphi \right)+\mathrm{ycos}\left(\varphi \right)\right],$$

where n_0_ represents the average refractive index of the volume grating, Δn is the RIM of the holographic recording material, Λ is the grating period, and φ is the grating slanted angle of VHG.

## 3. Results and Discussion

### 3.1. Holographic Performances Influenced by Exposure Parameters

**Figure 4:**
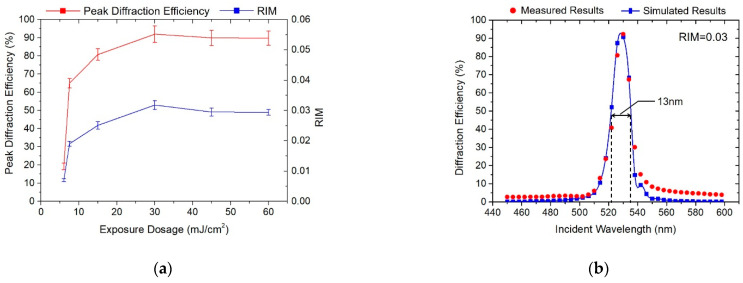
Holographic properties influenced by different exposure dosage. (**a**) Maximum diffraction efficiency and RIM curves of gratings under different exposure dosage; (**b**) Optimized diffraction efficiency curves ranging from 450 nm to 600 nm.

Firstly, we examined the influences of exposure dosage on the holographic properties of the VHG samples. To compare the holographic performances under different exposure dosages, the photopolymer was illuminated under the same light intensity and prepared under similar post-treatment conditions. The exposure intensity was set at 10 mW/cm^2^, and the dark reaction time, UV curing time, baking time and temperature were 4 min, 3 min, 5 min and 100 °C, respectively. With the increment of exposure dosage, all the peak diffraction efficiencies of samples underwent an obvious promotion, as shown in Figure 4. In particular, when the exposure dosage reached the initial exposure thresholds, the measured diffraction efficiency increased rapidly. When the diffraction efficiency reached over 60%, the growth slowed down until reaching the maximum value. Finally, the peak diffraction efficiency remained almost the same, even if the exposure dosage kept increasing.

The photo-polymerization reaction mechanism was well demonstrated by the experimental results. At initial exposure, the photosensitizer was continuously consumed, initiating monomer polymerization. The concentration of the photosensitizers and monomers in the bright region decreased continuously, forming a huge concentration difference between bright and dark regions. Due to the concentration gradient, the photosensitizers and monomers in the dark regions form a refractive index modulation, so the diffraction efficiency increased rapidly. With the progress of the reaction and polymerization, concentration differences of components between bright and dark regions decreased gradually, thus slowing down the increase in diffraction efficiency.

In addition, the exposure dosage that resulted in the peak diffraction efficiency of the VHGs was only 30 mJ/cm^2^, which means that the light sensitivity of acrylate photopolymer is very high. We can also see that the peak diffraction efficiency reached 92%, the maximum diffractive wavelength bandwidth reached around 13 nm, and the corresponding RIM was 0.03.

**Figure 5:**
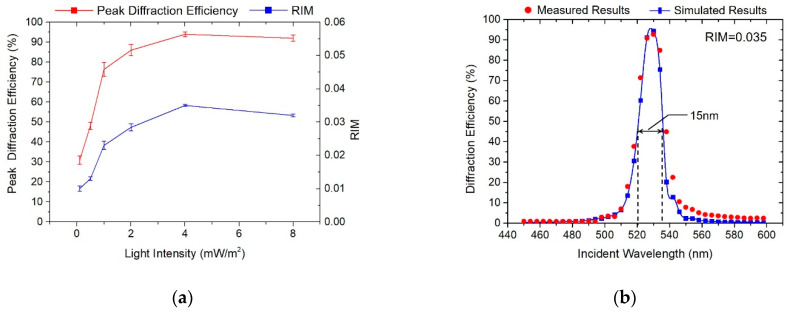
Holographic properties influenced by different light intensities. (**a**) Peak diffraction efficiency and RIM evolution curves of gratings; (**b**) Optimized diffraction efficiency curves.

Similarly, samples were illuminated under the same exposure dosage (30 mJ/cm^2^) and post-treatment with different light intensities, as shown in Figure 5. As is illustrated, the diffraction efficiency was enhanced with the increase of exposure intensity (from 0.1 mW/cm^2^ to 8 mW/cm^2^). However, the peak diffraction efficiency changed significantly when the light intensity was below 1 mW/cm^2^. In general, photo-polymerization occurs only when the recording light intensity reaches a certain threshold [8]. This is because the photosensitizer has a strong absorption at 533 nm when the incident light intensity is too low, and most of the light will be absorbed by the material. Only when the light intensity exceeds a certain threshold (1 mW/cm^2^), will the material be photoinduced to polymerize. On the other hand, low light intensity means that it is necessary to extend the exposure time, and then the environmental vibration and temperature changes will affect the exposure quite a lot. Additionally, the higher the intensity of the light exposure, the more photons enter the materials within a certain period of time, leading to an increase in the photoinduced reaction rate and mutual diffusion rate between the photosensitizers and the monomers. Therefore, the light intensity also affects the polymerization speed of the photopolymers. After further optimizing the light intensity, the peak diffraction efficiency, diffractive wavelength bandwidth and RIM increased to about 94.4%, 15 nm and 0.035, as shown in Figure 5.

### 3.2. Holographic Performances Influenced by Post-Treatment

**Figure 6:**
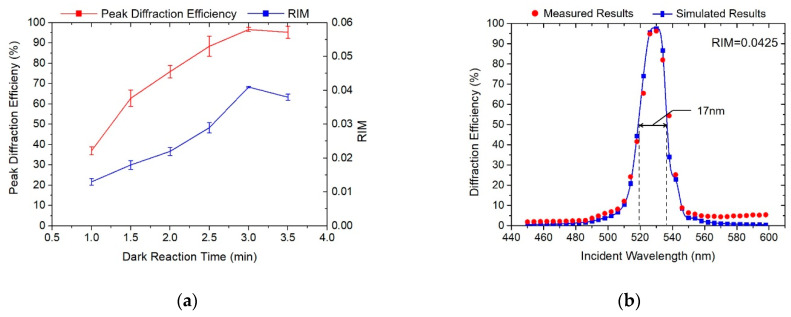
Holographic properties influenced by dark reaction time. (**a**) Peak diffraction efficiency and RIM evolution curves of gratings; (**b**) Optimized diffraction efficiency curves.

In addition to the exposure parameters, the post-treatment process also has a great impact on the holographic performance of the VHGs. When the exposure process is over, the photopolymerization and diffusion process do not stop immediately, and some reacted monomers will continue transferring, polymerizing and diffusing [28,29]. Therefore, in order to ensure the monomers fully polymerize and diffuse, the material needs to be placed in a dark room for a period of time after exposure. From the figure, it can also be seen that dark reaction time had a significant effect on the improvement of the grating diffraction efficiency within the first 2 min. When the dark reaction time exceeded 3 min, the dark reaction did not significantly improve the holographic performance of the VHG. As shown in Figure 6, with the increase in dark reaction time, the grating peak diffraction efficiency increased from 37% to 96.5%, the diffractive wavelength bandwidth also expanded to around 17 nm and the corresponding RIM reached 0.0425.

**Figure 7:**
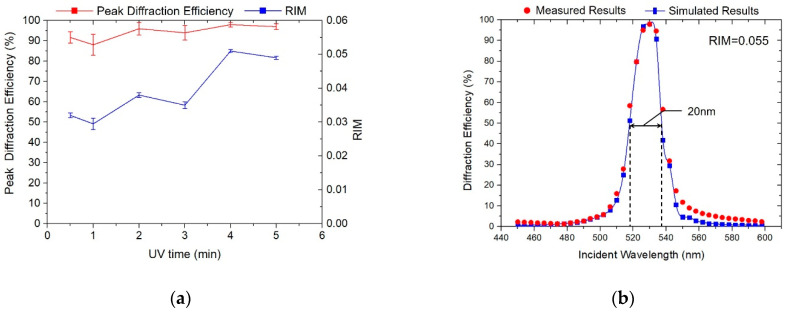
Holographic properties influenced by UV curing time. (**a**) Peak diffraction efficiency and RIM evolution curves of gratings; (**b**) Optimized diffraction efficiency curves.

According to the Ref. [30], the UV curing should consume the unreacted monomers. In this section, we characterize the influence of UV curing time on the holographic performance of VHG. On the basis of the above analysis, the exposure intensity and dosage were 4 mW/cm^2^ and 30 mJ/cm^2^. The dark reaction time was controlled at 3 min, the UV curing time was changed from 0.5 min to 5 min, and the other post-treatment parameters remained the same. As shown in Figure 7, the change in UV curing time had no significant effect on the peak diffraction efficiency. Considering the preparation efficiency of the holographic waveguide applied VHGs, we decide to decrease the UV curing time from 3 min to 2 min, under the premise of ensuring grating diffraction efficiency.

**Figure 8:**
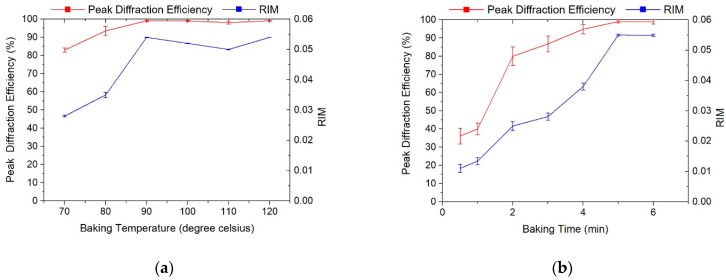
Peak diffraction efficiency and RIM evolution curves of gratings influenced by (**a**) baking temperature and (**b**) baking time.

After UV curing, a baking process is usually applied to the sample to enhance the peak diffraction efficiency and RIM of the photopolymer [30]. To investigate the effects of baking temperature on the RIM, we treated samples at several baking temperatures (from 70 °C to 120 °C, in interval of 10 °C) for baking times of 5 min. Figure 8a shows the experimental results of peak diffraction efficiency, and thus the RIM, as a function of baking temperature for a baking time of 5 min. In general, the optimum baking temperature was 90 °C, and the peak diffraction efficiency increased from 83.02% to 99.1%.

On the other hand, we also treated samples at several different baking times (from 1 min to 6 min, at intervals of 1 min) at a baking temperature of 90 °C. Figure 8b shows the experimental results of peak diffraction efficiency, and thus the RIM, as a function of baking time at the optimum baking temperature. It can be seen that the baking time has a great influence on the experimental results. Finally, when the baking time was below 2 min, the peak diffraction efficiency increased rapidly from 39% to 80%, and the corresponding RIM also improved from 0.01 to 0.025. At baking times of more than 2 min, although the peak diffraction efficiency continued to increase, the speed of increase was significantly slower. Until a baking time of 5 min, the results remained almost unchanged. Therefore, the best baking time was determined to be 5 min for a baking temperature of 90 °C.

**Figure 9:**
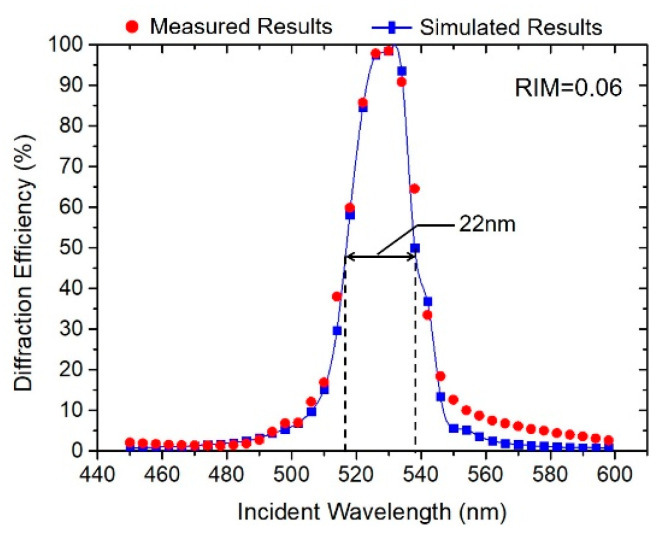
Measured diffraction efficiency curves of waveguide applied VHGs under optimum preparation conditions.

On the basis of the above optimization process, we prepared the monochromatic green holographic waveguide using the optimum exposure and post-treatment parameters (the exposure intensity was set as 4 mW/cm^2^, the dark reaction time, UV curing time, baking time and temperature were 3 min, 2 min, 5 min and 90 °C). After testing, the diffraction efficiency and diffractive wavelength bandwidth of the waveguide applied VHGs reached 99% and 22 nm, and the corresponding RIM reached 0.06, as shown in Figure 9.

On the basis of the experimental results, it was found that exposure dosage determines the degree of polymerization in the process of holographic recording, leading more monomers with small molecular weight to polymerize into chains. Exposure intensity determines the consumption rate of photosensitizer and polymerization rate of monomers, improving the speed of the VHG formation. By analyzing the effects of the post-treatment process, we found that the excited writing monomers could completely diffuse and polymerize into chains during the dark reaction time. In addition, UV curing irreversibly bleached the residual initiator dyes and cured binders, which helped to fix the formed grating structure. Then, the polymerized monomers needed to be cured by baking, forming a refractive index modulation between the light curing and the thermosetting of the monomers.

### 3.3. Display Results of Monochromatic Green Holographic Waveguides

**Figure 10:**
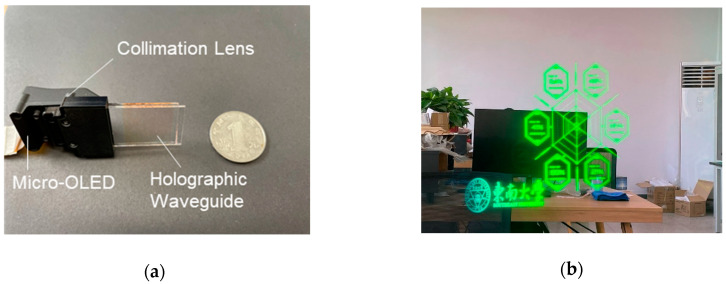
Realization of monochromatic green holographic waveguide display. (**a**) Display module picture; (**b**) Actual display images.

As shown in Figure 10, the green monochromatic holographic waveguide display module consisted of three components: a single green micro-OLED display (0.39′, Guozhao Optoelectronics Corporation, Nanjing, China), our own designed and fabricated collimation lens, and a holographic waveguide. In this compact display module, the micro-OLED provides a bright green image source, and the collimation lens is used to collect and collimate the lights emitted from the micro-OLED into the in-coupling VHG of the holographic waveguide. The holographic waveguide performed the role of diffraction imaging. The in-coupling VHGs diffract the collimating lights into the waveguide under the total inner reflection(TIR )condition, and then the diffracted lights propagate in the waveguide and diffract again through the out-coupling VHG into the human eyes.

The luminance of the micro-OLED and the holographic waveguide display module are around 10,000 cd/m^2^ and 300 cd/m^2^, respectively, and were measured using a point luminance meter CS 200, so the optical efficiency of the whole system is about 3%. The diagonal FOV and the eye relief were designed to be 28° and 15 mm. The display results are shown in Figure 10b and Video S1; it can be seen that the holographic waveguide is able to display green images very well in the real world after optimizing the preparation parameters.

**Figure 11:**
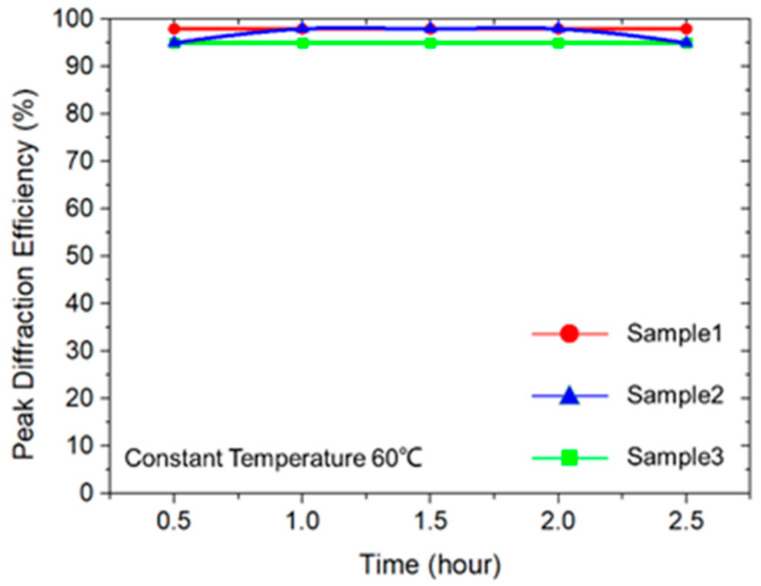
The diffraction evolution curve of three samples baking for 2.5 h at 60 °C.

To verify the thermal stability of the prepared holographic waveguide, we baked three samples in an oven at a constant temperature of 60 °C for 2.5 h. The experimental results show that the diffraction efficiency of the VHGs remained almost unchanged, as shown in Figure 11.

## 4. Conclusions

In this paper, we investigated the optimal conditions of a green light-sensitive acrylate-based photopolymer, and a monochromatic green holographic waveguide was fabricated to verify its potential application in an AR diffractive waveguide.

In the optimization research, the influence of the exposure parameters and post-processing conditions on the diffraction performance were investigated. We successfully increased the polymerization speed and degree of photopolymerization by adjusting the exposure intensity, exposure dosage, baking time, and baking temperature.

We found that, as the exposure dosage increased, the diffraction efficiency quickly increased to 60%, then the increasing speed of diffraction efficiency became slow, although the exposure dosage was continually increasing. On the other hand, we studied the threshold of exposure intensity and found that when the light intensity was lower than 1 mw/cm^2^, a higher diffraction efficiency could not be achieved.

Additionally, in the post-processing stage, proper dark-reaction and UV curing were able to effectively improve the holographic performances of VHGs. A dark reaction of 2 min was able to improve the diffraction efficiency significantly. Compared with the other preparation parameters, the UV curing time had no obvious effect on the peak diffraction efficiency. The peak diffraction efficiency increased slowly with the increment of baking temperature, and when the baking temperature was 90 °C, the efficiency reached a maximum of 99.1%. A baking time of 2 min was able to rapidly increase the diffraction efficiency from 39% to 80%, while the corresponding RIM also improved from 0.01 to 0.025. After 2 min, the growth slowed down until reaching its maximum value.

It was also found that when the diffraction efficiency reached its maximum value of 99%, the diffractive wavelength bandwidth still continuously increased with increasing RIM. When the RIM of VHGs reached 0.06, with an exposure dosage of 30 mJ/cm^2^, the corresponding diffractive wavelength bandwidth reached around 22 nm, proving that the acrylate-based photopolymer had a good capacity with wide FOV and high optical efficiency for holographic waveguide display.

To verify the application potential of this material in waveguide displays, we prepared a monochromatic green holographic waveguide and further developed a compact AR display system. A monochromatic green near-eye display with FOV 28° was achieved, and the display results proved that this photopolymer can be regarded as a high-performance holographic recording material that could find wide application in the field of waveguide display.

## Figures and Tables

**Table 1:** Composition of the acrylate photopolymer in wt%.

Component	Concentration (wt%)
polyvinyl acetate	55.4
NVC	23.5
tetrahydrofurfuryl acrylate	9.8
2-phenoxyethy acrylate	6.1
BCIM	5.14
EB	0.02

## Data Availability

The data presented in this study are available on request from the corresponding author.

## Supplementary Materials

The following are available online at https://www.mdpi.com/2073-4360/13/6/936/s1, Video S1: Monochromic green imaging results of holographic waveguide display system.
